# Endovascular Treatment of Congenital Internal Carotid-Jugular Fistula

**DOI:** 10.3389/fneur.2018.01118

**Published:** 2018-12-18

**Authors:** Ming Wang, Weijian Fan, Rajneesh Mungur, Jun Gu, Shu Wan

**Affiliations:** ^1^Department of Neurosurgery, The First Affiliated Hospital, College of Medicine, Zhejiang University, Hangzhou, China; ^2^Brain Center, Zhejiang Hospital, Hangzhou, China

**Keywords:** internal carotid-jugular fistula, internal carotid artery, jugular vein, endovascular treatment, stent

## Abstract

A carotid-jugular fistula is a direct communication between the carotid artery and the jugular vein. Both congenital and spontaneous internal carotid-jugular fistulas are extremely rare. We describe the first case of successful endovascular treatment for a congenital internal carotid-jugular fistula. We report a 64-year-old woman who presented with a pulsatile mass swelling over the left cervical region and right hemiparesis after cough. Digital subtraction angiography confirmed the diagnosis of left high-flow internal carotid-jugular fistula. The fistula was successfully treated by fractional stent-assisted embolization.

## Introduction

A carotid-jugular fistula is a direct communication between the carotid artery and the jugular vein, and usually reported in children (1, 2). Congenital and spontaneous internal carotid-jugular fistulas are extremely rare (3, 4). Because of its rarity, there is little information about treatment of such disease. Here, we reported a 64-year-old woman who presented with a pulsatile mass swelling over the left cervical region and right hemiparesis after cough. Digital subtraction angiography (DSA) revealed a left high-flow internal carotid-jugular fistula. After fractional stent-assisted embolization, the fistula was totally occluded, and the patient recovered well. This case provides a successful endovascular treatment for the internal carotid-jugular fistula.

## Case Presentation

**Figure 1:**
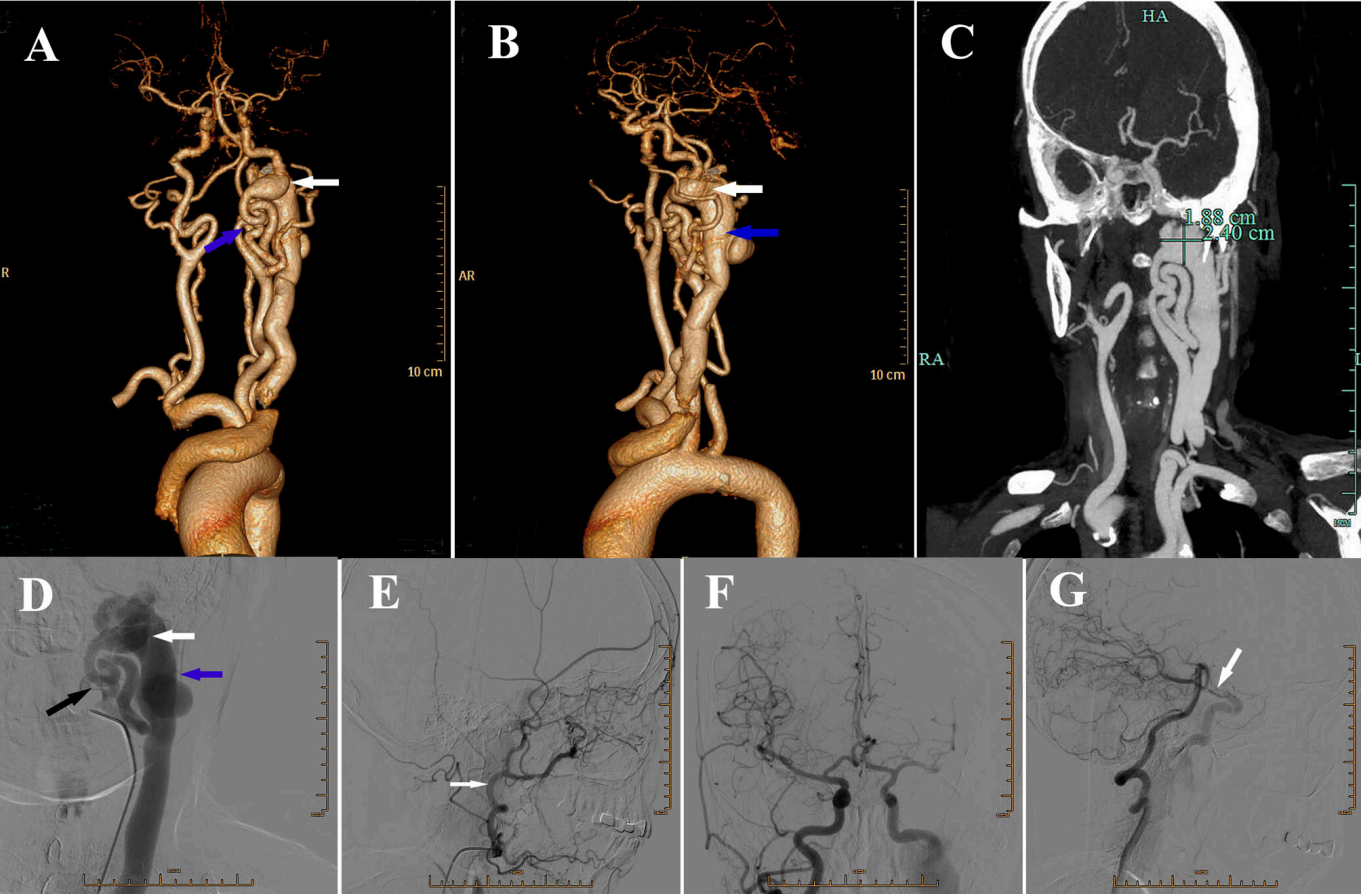
**(A)** Anteroposterior view of three-dimensional volume rendering of computed tomography angiography (CTA) showing a fistula (white arrow) located at the lever of craniocervical junction, with severe vascular distortion (blue arrow) at the beginning of left internal carotid artery (ICA). **(B)** Lateral view of three-dimensional volume rendering of CTA showing the dilatation of internal jugular vein (blue arrow). White arrow: the fistula. **(C)** CTA showing the size of fistula. **(D)** Digital subtraction angiography (DSA) showing the fistula (white arrow), dilatation of internal jugular vein (blue arrow) and the absence of blood flow from left ICA (black arrow) into the brain. **(E)** DSA of left external carotid artery (ECA) (white arrow) showing no obvious abnormality. **(F)** DSA showing right ICA supplying the left anterior cerebral artery and middle cerebral artery via the anterior communicating artery. **(G)** DSA showing vertebral basilar artery supplying contralateral cerebral via left posterior communicating artery (white arrow).

A 64-year-old woman was admitted to our hospital with a pulsatile mass swelling over the left cervical region and right hemiparesis after cough for 1 day. She had a history of fibromatosis, but no previous history of trauma, operation or inflammation in this region. On physical examination, a 5-cm large pulsatile swelling with blowing bruit was found over the left cervical region and multiple fibromas were found in regions of face, neck, and trunk. Neurological examination revealed muscular strength of grade IV according to the ‘manual muscle test (MMT)' grading system and hypoesthesia of right limbs, including touch and pain sensation. Computed tomography angiography (CTA) and DSA revealed a left high-flow internal carotid-jugular fistula at the first cervical level and twisted left internal carotid artery (ICA) (Figures 1A–D). Blood flow from the vertebrobasilar artery and right ICA via the circle of Willis supplied the left anterior cerebral artery and middle cerebral artery and drained backwards into the petrosal segment of left ICA (Figures 1E–G).

**Figure 2:**
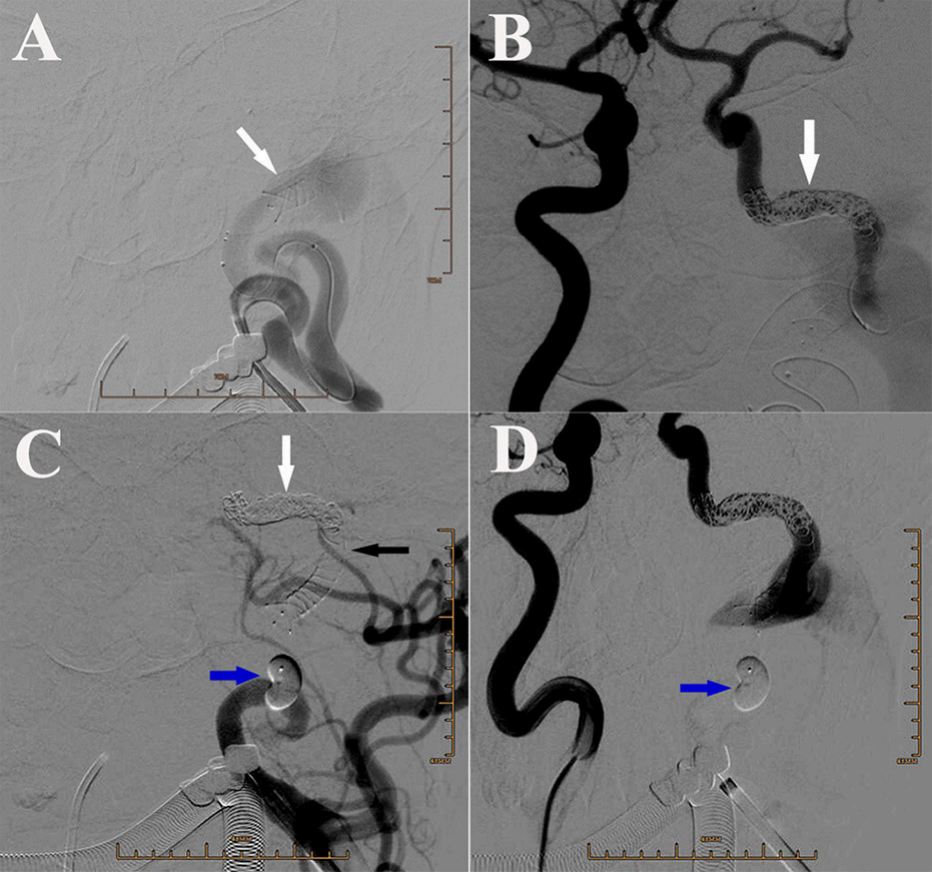
**(A)** DSA of left ICA showing the placement of the LVIS stent (white arrow) and the Echelon microcatheter. **(B)** DSA of right ICA showing the backward blood flow in the distal part of fistula that was reduced after coiling. White arrow: coils. **(C)** DSA of left ICA showing placement of one detachable balloon (blue arrow) to block the ICA near the proximal part of fistula. White arrow: coils. Black arrow: stent. **(D)** DSA of right ICA showing placement of one detachable balloon (blue arrow) to block the ICA near the proximal part of fistula.

We chose embolization of both the fistula and parental artery, because no appropriate covered stent could be used to pack the fistula in the condition of reservation of left ICA. The purpose of first endovascular treatment was to isolate the fistula, by blocking both the backward blood flow from right ICA and the forward blood flow from left ICA. A Headway-21 stent catheter was selectively inserted into the distal part of fistula in the segment of carotid cavernous sinus, meanwhile an Echelon-10 microcatheter was placed in the distal part of stent catheter. One LVIS 5.5^*^30 mm stent, with its characteristic relatively compact mesh, was used to cover the distal part of fistula to avoid coils being pushed into internal jugular vein and heart (Figure 2A). However, the 3.5 cm fistula could not be covered by the LVIS stent. Therefore, 10 coils were additionally used one by one, to block the fistula from the distal to proximal part through an Echelon-10 microcatheter (Figure 2B). When considering the fast-backward blood flow from the right ICA after embolization, we could not guarantee the safety of blocking by Onyx. We chose one detachable balloon to block the ICA near the proximal part of fistula to stop the forward blood flow, and the backward blood flow would be stopped in the second treatment (Figures 2C,D).

**Figure 3:**
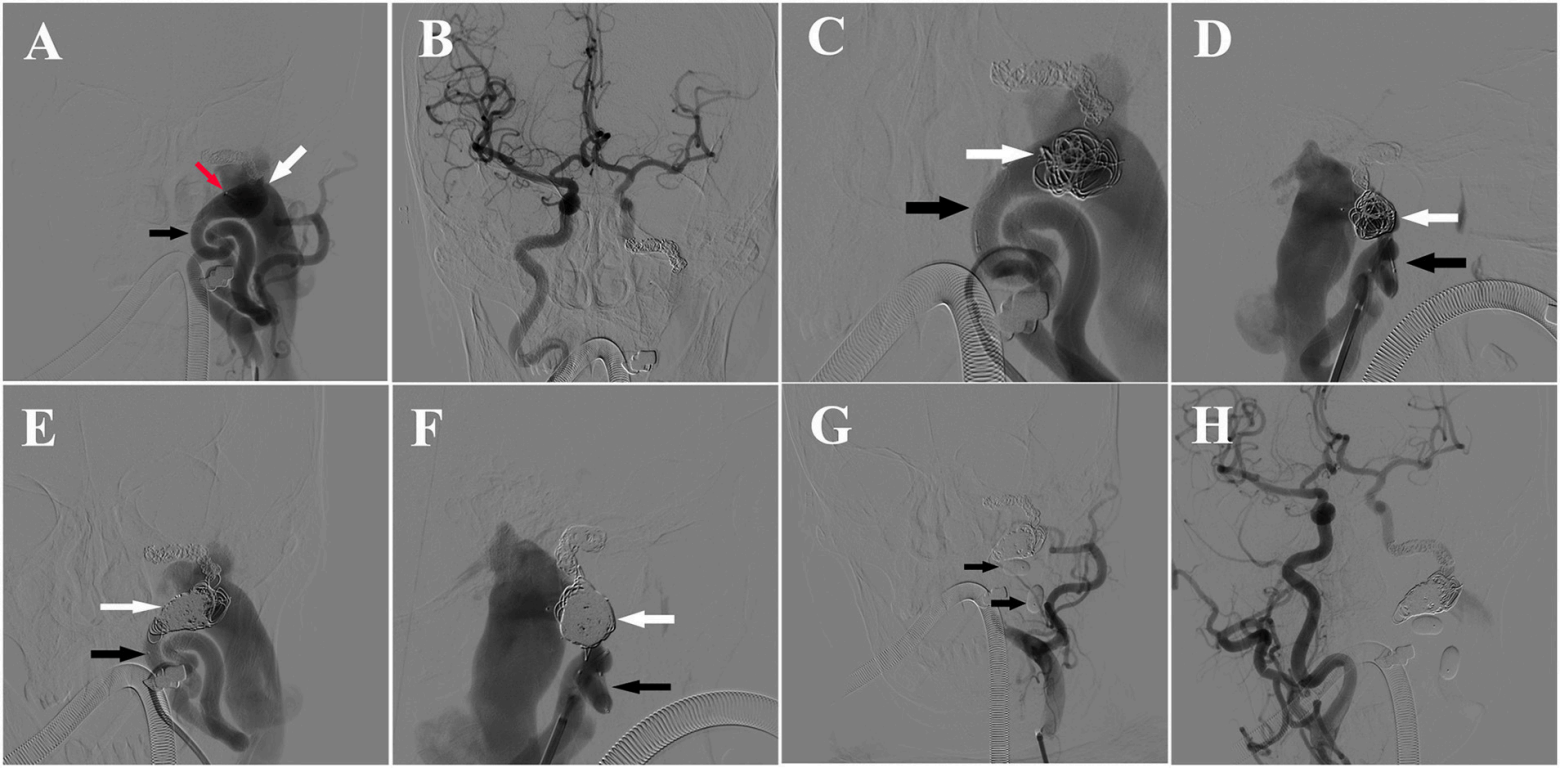
**(A)** DSA of left ICA showing the balloon (red arrow) in the ICA (black arrow) near the fistula and residual blood flow in the fistula (white arrow) from the left ICA. **(B)** DSA of right ICA showing backward blood flow from the right ICA. **(C)** Anteroposterior view of DSA showing the fistula embolization with Double-microcatheter techniques. Black arrow: ICA, white arrow: coils in the fistula. **(D)** Lateral view of DSA showing the fistula embolization with Double-microcatheter techniques. Black arrow: ICA, white arrow: coils in the fistula. **(E)** Anteroposterior view of DSA of left ICA showing obvious forward blood flow from the left ICA (black arrow) to fistula after embolization (white arrow). **(F)** Lateral view of DSA of left ICA showing obvious forward blood from the left ICA (black arrow) to fistula after embolization (white arrow). **(G)** DSA showing placement of two Balt detachable balloons (black arrows) in the proximal part of fistula to block the blood flow of left ICA. **(H)** DSA of right ICA showing the backward blood flow was reduced significantly after the fistula coiling.

The blowing bruit was reduced significantly after the first treatment, but became worse after 2 months later. The patient was admitted to our hospital again, and DSA revealed residual blood flow in the fistula from the left ICA and premature balloon deflation (Figures 3A,B). It was fortunate that LVIS stent blocked the balloon into the internal jugular vein. An additional 13 coils were used to pack the fistula with double-microcatheter techniques, under the multi-angle DSA projection, to avoid the coil protruding into the internal jugular vein (Figures 3C–F). Afterwards, two detachable balloons were used to block the proximal part of left ICA again (Figure 3G). DSA revealed the forward blood flow disappeared, the low-flow backward blood from right ICA still supplied the fistula, and a normal ipsilateral jugular vein (Figure 3H). Because the fistula was mostly blocked, and the low-flow backward blood may promote the formation of thrombosis in the distal part of fistula, we stopped the second endovascular treatment and planed a DSA examination to evaluate the effect of embolization and to determine further treatment. After this treatment, the patient did not feel the blowing bruit or any other discomfort.

**Figure 4:**
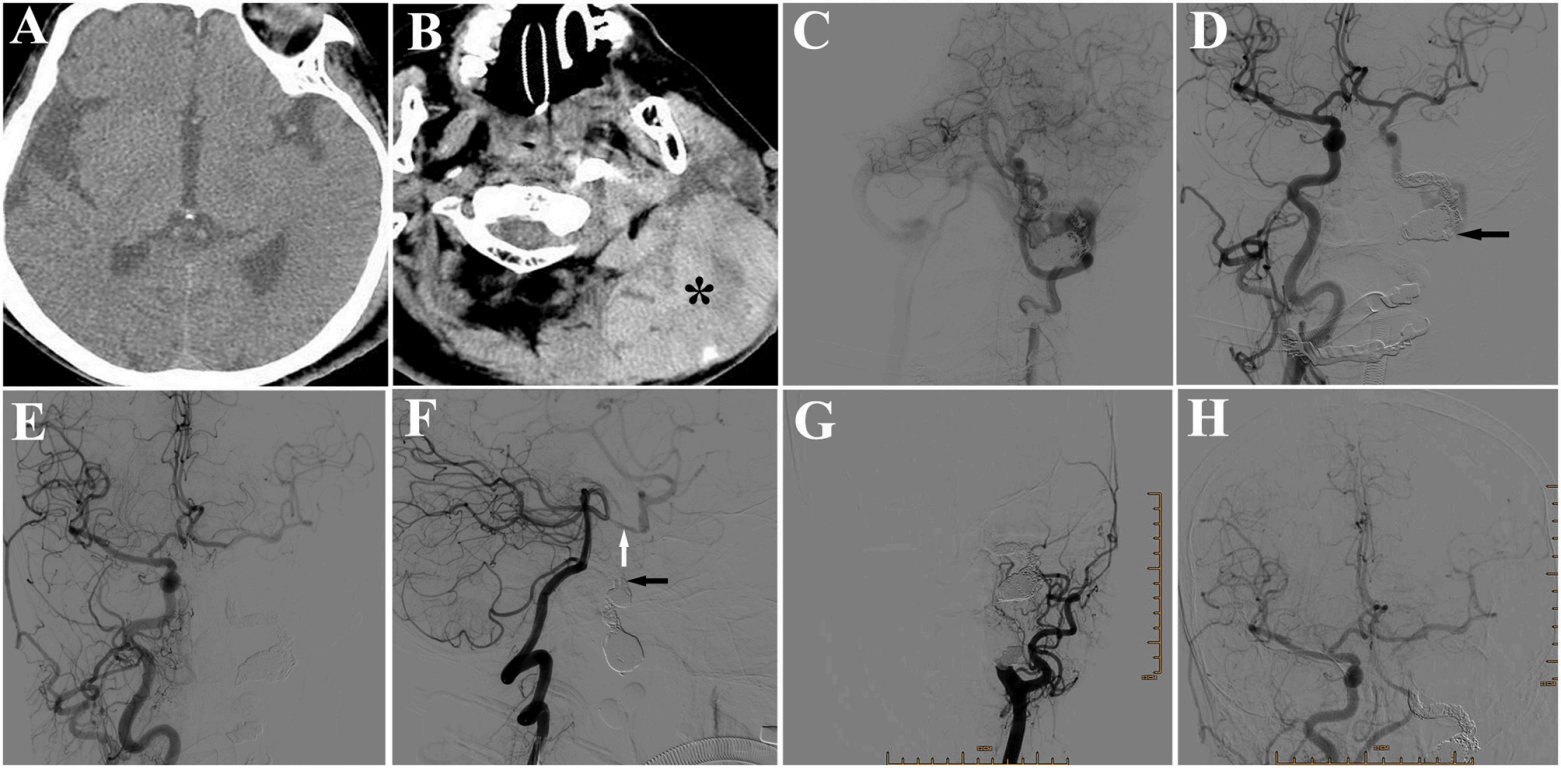
**(A)** CT showing no abnormality in the brain. **(B)** CT showing a subcutaneous hematoma (*) in left occipitocervical region. **(C)** DSA of left vertebral artery showing little backward blood flow in the fistula from the posterior communicating artery, but no appearance of internal jugular vein in the proximal part of the fistula. **(D)** DSA of right ICA showing little backward blood flow in the fistula from the anterior communicating artery, but no appearance of internal jugular vein in the proximal part of the fistula (arrow). **(E)** DSA of right common carotid artery (CCA) showing fistula disappearance after placement of 3 coils and a 1.2 ml Onyx-18. **(F)** DSA of left vertebral artery showing no backward blood flow in the fistula (black arrow). White arrow: posterior communicating artery. **(G)** Six-month follow-up DSA of left CCA showing a totally occluded fistula, in 6-month follow-up. **(H)** DSA of right CCA showing a totally occluded fistula.

However, the patient complained of swelling and pain in the left occipitocervical region 2 days later. Physical examination revealed a large subcutaneous mass, with volatility and severe tenderness. Emergent CT revealed a subcutaneous hematoma in left occipitocervical region, without abnormalities in brain (Figures 4A,B). DSA in the hybrid operation room showed the coils in the fistula were stable without no forward blood flow in the proximal part of fistula. However, there was still a little backward blood flow through the right ICA and vertebrobasilar artery into right internal jugular vein, via the distal part of fistula. The lower part of fistula was not shown, because the blood drained into the right internal jugular vein through the sigmoid and transverse sinus reversely (Figures 4C,D). It was suggested that the occipitocervical subcutaneous hemorrhage was caused by poor ipsilateral jugular drainage, which was the result of the formation of thrombosis in fistula extending to the left internal jugular vein, thus blocking forward and backward reflow. We inserted an Echelon microcatheter through the left vertebrobasilar artery-posterior communicating artery, then selectively to petrosal segment of the left ICA, where 3 coils were put in, and then a 1.2 ml Onyx-18 was injected slowly. DSA examination after this procedure revealed that the backward blood flow distal to the fistula disappeared (Figures 4E,F). An open operation to remove the hematoma was preformed after the interventional operation. Multiple capillary hemorrhages were found from behind the sternocleidomastoid muscle after removing clots. The operation to stop bleeding was successful, and the postoperative course was uneventful. A 6-month follow-up DSA demonstrated a totally occluded fistula allowing the patient to engage in light manual labor with normal neurological functioning after the operation (Figures 4G,H).

## Discussion

Most of carotid-jugular fistulas are acquired and typically caused by either a penetrating trauma to the cervical region, iatrogenic trauma, or phlegmona in the laterocervical regions. Spontaneous and congenital carotid-jugular fistulas are extremely rare, with only a limited number of cases reported in the literature. Of these, only two have a fistula between the internal carotid artery and the internal jugular vein. The first case of congenital internal carotid artery resolved spontaneously (3), and the other case was treated with surgical repair of arteriovenous communication (4). Herein, we reported the third case of congenital internal carotid-jugular fistula as well as the first case of successful endovascular treatment for a congenital internal carotid-jugular fistula.

Congenital carotid-jugular fistulas were usually reported in children (1, 2), but the patient in this case had the first symptom at the age of 64. Pulsatile mass swelling is the main clinical manifestation. However, carotid-jugular fistulas are potentially dangerous, because they may cause serious complications of cardiac failure, rupture, thromboembolism, or cerebral ischemia (5), like hemiparesis in our case.

For its rarity, neither appropriate procedural standards or definitive surgical techniques have yet been established to treat congenital internal carotid-jugular fistulas. Operative ligations, detachable balloons, coiling, and stenting were all reported as treatment options for carotid-jugular fistulas (1, 4, 5). Operative ligation was not appropriate for our case, because of its location in the cranio-cervical region. Because of difficulty to expose the entire fistula and high risk of bleeding and nerve injury with invasive surgical open repair, we chose endovascular treatment for this patient. The length of 3.5 cm and the characteristic high-flow made our treatment more challenging. Simple coiling has no role in the high-flow internal carotid-jugular fistula, but has risk of iatrogenic pulmonary embolism or cardiac failure if the coiling was pushed out of the fistula. Although, there is no long enough stent to over the entire fistula. Therefore, stent-assisted coiling was applied. A stent was placed in the distal part of fistula to prevent coiling going out, and embolization with coils was proceeded from the distal part. Additionally, we use two balloons to block the proximal part of fistula after coiling to reduce the blood flow from the ICA. For the high-flow blood, we could not make a total occlusion, however the blood flow was reduced obviously after the first endovascular treatment. Three months later, we proceeded with the second embolization and obliteration the fistula. However, due to the venous return being affected by the embolization, a subcutaneous hemorrhage onset 2 day post the second endovascular treatment. This should be noticed by neurosurgeons.

## Conclusion

We presented the first case of successful endovascular treatment for a congenital internal carotid-jugular fistula. The management of long, high-flow fistula is challenging. Stent-assisted coiling was suggested as an effective and safe treatment. Follow-up DSA was needed and neurosurgeons should be aware of both local hemorrhage and cerebral ischemia after endovascular treatment.

## Ethics Statement

This study was carried out in according with the recommendations of ‘Ethics committee of the First Affiliated Hospital, College of Medicine, Zhejiang University' with written informed consent from all subject. All subjects gave written informed consent in according with the Declaration of Helsinki.

## Author Contributions

MW was responsible for writing and figure organization. WF, JG, and SW were responsible for performance of endovascular treatment. RM was responsible for English editing. SW was responsible for conceptual input and manuscript review.

### Conflict of Interest Statement

The authors declare that the research was conducted in the absence of any commercial or financial relationships that could be construed as a potential conflict of interest.

## Abbreviations

CCA: common carotid artery
CJF: carotid-jugular fistula
CTA: computed tomography angiography
DSA: digital subtraction arteriogram
ECA: external carotid artery
ICA: internal carotid artery.
